# An evaluation of thyroid autoimmunity in patients with beta thalassemia minor: A case-control study

**DOI:** 10.12669/pjms.335.13210

**Published:** 2017

**Authors:** Ali Ramazan Benli, Sati Sena Yildiz, Mehmet Ali Cikrikcioglu

**Affiliations:** 1 Ali Ramazan Benli, Department of Family Medicine, Faculty of Medicine, Karabuk University, 78200, Karabuk, Turkey; 2 Sati Sena Yildiz, Department of Internal Medicine, Faculty of Medicine, Bezmialem Vakif University, 34093, Istanbul, Turkey; 3 Mehmet Ali Cikrikcioglu, Department of Internal Medicine, Faculty of Medicine, Bezmialem Vakif University, 34093, Istanbul, Turkey

**Keywords:** Anti-thyroid peroxidase, Anti-thyroglobulin, Beta thalassemia minor, Thyroid hormones

## Abstract

**Objective:**

The tendency to autoimmune diseases has been reported to be increased in beta thalassemia minor **(**BTM). The aim of this study was to evaluate whether thyroid autoimmunity is higher in BTM.

**Methods:**

Patients with BTM (n=86) and a healthy control group (n=93) were included in this cross-sectional case-control study. The two groups were compared in terms of anti-thyroglobulin (anti-TG) and anti-thyroid peroxidase (anti-TPO) and thyroid hormones.

**Results:**

In the BTM group, thyroid hormones and serum anti-TG and anti-TPO antibody levels were not statistically different from those of the control group. The BTM and control groups were similar in terms of anti-thyroid antibody (ATA) positivity prevalence. In the BTM group, anti-TG was 11.6% and anti-TPO was 14% positive, while these values were 14% and 12.9% positive, respectively in the control group (p=0.806 and p=0.989, respectively). The proportion of anti-TG and/or anti-TPO antibody positive subjects was found to be 20.9% in the BTM group, and 20.4% in the control group (p=0.919). The ratios of subjects with euthyroidism, hyperthyroidism and hypothyroidism were similar in both groups.

**Conclusions:**

As the thyroid autoimmunity prevalence in the BTM group was not increased compared to the control group, it can be considered that there is no necessity for routine ATA and thyroid hormone testing in subjects with BTM.

## INTRODUCTION

Beta Thalassemia Minor (BTM) is a chronic haemolytic anaemia appearing with hypochromic microcytic erythrocyte indexes and mild anaemia originating from impaired haemoglobin synthesis caused by a hereditary reduction in beta globin synthesis. BTM is prevalent in many regions of the world, including Mediterranean countries, the north coast of Africa, the Middle East, Central Asia, Southeast Asia, the Far East and South America. The highest BTM frequency is reported to be in Cyprus (14%), Sardinia (10.3%).^1^ Although thalassemia major, which is caused by deficient or a complete absence of production in beta globin synthesis, presents with quite a serious disease status, there is a widespread understanding that subjects with BTM do not have a significant problem in general.^1^,^2^ On the other hand, studies have reported that many diseases are seen more frequently in subjects with BTM than in people with no BTM.^3^ The risk of birth defects, gestational diabetes, diabetes mellitus Type-2, renal diseases, decreasing pulmonary functions, osteoporosis, dental problems, depression and fibromyalgia is increased in BTM patients.^4^-^10^ In addition, it has also been suggested that BTM creates a tendency to autoimmune disorders.^2^ The prevalence of BTM is significantly increased in rheumatoid arthritis; likewise, the incidence of rheumatoid arthritis is increased in BTM compared to the general population.^3^ Furthermore, systemic lupus erythematosus (SLE) has been reported to exhibit a more serious course in subjects with BTM.^11^ Rheumatological complications of BTM have also been reported.^3^ Free thyroxine (FT4) and thyroid stimulating hormone (TSH) have been found to be higher in BTM patients than in a normal group.^12^ It is not currently known whether the prevalence of thyroid autoimmunity is increased or not in BTM. The aim of this study was to evaluate thyroid autoimmunity in BTM.

## METHODS

This is a cross-sectional case-control study. It included a total of 86 patients with BTM who presented consecutively at the outpatient clinics of Bezmi-Alem Vakif University Internal Medicine Department, Istanbul, between October 2013 and January 2014. A healthy control group was formed of 93 participants who were age and gender-matched with study group and were presented consecutively at the outpatient clinics of the Internal Medicine department between November 2013 and January 2014. Informed consent was obtained from all participants.

### Inclusion criteria

Subjects 18-65 years of age with and without BTM were enrolled in the study.

### Exclusion criteria

Patients were excluded from the study for the following reasons: Gestation, lactation, iron deficiency anaemia, rheumatoid collagen tissue diseases, celiac disease, Type-1 diabetes, pernicious anaemia, stage 3 and further chronic kidney disease, chronic hepatitis, primary biliary cirrhosis, portal hypertension, idiopathic thrombocytopenic purpura, idiopathic hypoparathyroidism, Addison disease, lymphocytic hypophysitis, colitis ulcerosa, Crohn’s disease, Behcet’s disease, sarcoidosis, bronchial asthma, autoimmune haemolytic anaemia.

### Allocation of subjects to BTM and control group

Patients with mean corpuscular volume (MCV) <80fL, mean corpuscular haemoglobin (MCH) <27µg and Haemoglobin A2 (HbA2) ≥3.5% were considered to have BTM. Subjects with normal MCV, normal MCH and no anaemia (males: haemoglobin ≥13g/dL, females: haemoglobin ≥12g/dL) comprised the control group.

### Evaluation of anti-thyroid antibody positivity and definition of thyroid disorders

All patients with anti-TG and/or anti-TPO levels exceeding the upper limit of the reference range were considered ATA positive patients. A subclinical hypothyroidism diagnosis was established in the presence of normal FT4 and high TSH values, an overt hypothyroidism diagnosis in the presence of low FT4 and high TSH, and hyperthyroidism diagnosis with high FT4 and/or high free iodothyronine (FT3) and low TSH. The diagnosis of Graves’ disease required high FT4 and/or FT3, low TSH, clinical signs of hypermetabolic state diffuse activity increase in thyroid scintigraphy, diffuse thyroid enlargement in the ultrasound and presence of ophthalmopathy. In this study, ATA positive patients and Graves’ disease are referred to with the term thyroid autoimmunity.

### Evaluation of patients

For each participants enrolled in the study, disease history, personal history and family history were taken; medication use and smoking status were queried, and a systemic physical examination was performed. Comorbidities were determined from blood and urine analysis and imaging techniques (when necessary).

### Laboratory Tests

Blood samples were obtained in the early morning after overnight fasting. Blood analysis was performed on the same day. Complete blood cell analysis was performed with a Sysmex XT 1800i (ROCHE-2011, Kobe, Japan) device. Biochemical assays were performed on a COBAS 8000 (ROCHE-2007, Tokyo, Japan) device using COBAS-C system kits.

Hb electrophoresis was performed using a Shimadzu 20-A device (Shimadzu-2013, Kyoto, Japan) with the high prominence liquid chromatography (HPLC) method.

Thyroid hormones, anti-TG and anti-TPO antibodies were examined on a Siemens Advia Centaur device (Sıemens-2006, Dublin, Ireland) with the chemiluminesence method using Advia Centaur (Advia-2013-Tarrytown, USA) kit.

### Statistics

Numerical variables were presented as mean with standard deviation, and nominal variables in ratios. Subjects recruited to the study were classified into two groups as the BTM group and the control group. Nominal independent variables such as gender, smoking, ATA positivity, etc. were compared between the groups using the Chi-square test. One sample Kolmogorov-Smirnov test was applied to determine if the continuous (numerical) independent variables were normally distributed. Normally distributed independent continuous variables were compared between the groups with the Student’s t-test, and non-normally distributed independent continuous variables were compared with Mann-Whitney U-test. Bivariate correlation test was also performed. Two tailed p value<0.05 was considered statistically significant.

## RESULTS

The study comprised 86 patients with BTM and 93 control subjects with a mean age of 41.34 ±13.09 years. The BTM group comprised 42 females and 44 males with a mean age of 40.52±13.11 years. The control group comprised 46 females and 47 males with a mean age of 42.21±13.26 years. Age and gender distribution were determined to be similar in the BTM group and the control group (Tables I and II). The BTM group and the control group were also comparable in respect of comorbidities and smoking status (Table-II).

**Table-I:** Comparison of demographics and blood analysis findings of two groups.

*Variable*	*BTM (n=86)* *Mean ± SD*	*Controls (n=93)* *Mean ± SD*	*Reference value*	*P-value*
Age, years	40.52 ± 13.11	42.21 ± 13.26		0.411
RBC x 10^12^/L	6.27 ± 0.71	5.03 ± 0.39	4.0-6.2	<0.001
Hb, g/L	120.5 ± 12.4	139.4 ± 14.0	130-175	<0.001
Htc, %	37.60 ± 3.60	42.09 ± 3.40	40-52	<0.001
MCH, pg/cell	18.77 ± 0.80	29.22 ± 1.62	27-34	<0.001
MCV, fL	60.57 ± 3.68	84.17 ± 3.89	80-100	<0.001
RDW, %	17.53 ± 0.84	13.01 ± 0.53		<0.001
WBC, x10^9^/L	7.52 ± 1.84	6.98 ± 1.78	3.8-10.0	0.053
Platelet, x10^9^/L	274.83 ± 63.48	246.43 ± 57.57	150.0-400.0	0.003
HbA2, %	5.31 ± 0.89		1.5 - < 3.5	
HbA, %	91.38 ± 5.35		95 - 98	
HbF, %	0.93 ± 0.99		<2	
ESR, mm/h	9.74 ± 7.75	12.5 ± 10.75	<20	0.122
CRP, nmol/L	0.57 ± 1.65	0.41 ± 1.03	0-0.48	0.404
Glucose, mmol/L	5.63 ± 1.27	5.9 ± 2.0	3.9-5.55	0.462
HbA1C, %	5.40 ± 0.59	5.63 ± 1.01	4.5-5.7	0.331
TSH, mIU/L	2.01 ± 2.37	2.00 ± 2.02	0.63-4.82	0.443
FT3, pmol/L	5.21 ± 3.75	4.89 ± 0.51	3.5-6.5	0.582
FT4, pmol/L	15.03 ± 7.61	14.35 ± 2.17	10-18.7	0.878
Anti TG, IU/mL	50.70 ± 13.37	44.29 ± 92.58	0-64	0.161
Anti TPO, IU/mL	112.68 ± 32.00	117.00 ± 28.03	0-57	0.641

RBC: Red blood cell, RDW: Red cell distribution widht, HbA: Haemoglobin A, HbA1C: Haemoglobin A1C, HbF: Haemoglobin F, ESR: Eritrocyite sedimentation rate, CRP: C-Reactive protein.

**Table-II:** Gender, smoking status and comorbidities of study subjects.

*Variable*	*BTM (n=86)* *Number (%)*	*Controls (n=93)* *Number (%)*	*P value*
Gender, female/male	42/44 (48.8/51.2)	46/47 (49.5/50.5)	0.947
Diabetes mellitus Type-2	12 (14)	11 (11.8)	0.841
Essential hypertension	15 (17.4)	14 (15.1)	0.818
Chronic ischemic heart disease	1 (1.2)	1 (1.1)	0.414
Smokers	26 (30.2)	26 (28)	0.865
Ex-smokers	1 (1.2)	3 (3.2)	0.669

In the BTM group, haemoglobin (Hb), haematocrit (Htc), MCV and MCH values were lower (p<0.001, p<0.001, p<0.001 and p<0.001 respectively), and red cell distribution width and platelet count were higher (p<0.001 and p=0.003) compared to the control group. White blood cell counts were similar in the two groups (p=0.053) (Table-I).

The BTM group and the control group were comparable in respect of serum TSH, FT3, and FT4 levels (p=0.44, p=0.58 and p=0.87 respectively) (Table-I). In the BTM group serum anti-TG and anti-TPO levels were not statistically different from those of the control group (p=0.16 and p=0.64 respectively) (Table-I). The two groups were similar in respect of the frequency of anti-TG antibody and anti-TPO antibody positivity (p=0.80 and p=0.98 respectively) (Table-III). The proportion of anti-TG antibody and/or anti TPO antibody positive subjects were found to be similar in the two groups (p=0.91) (Table-III).

**Table-III:** Thyroid status and treatments of groups.

*Variable*	*BTM (n=86)* *Number (%)*	*Control (n=93)* *Number (%)*	*P-value*
Previous partial thyroidectomy	4 (4.7)	2 (2.2)	0.608
Anti-TG positivity	10 (11.6)	13 (14)	0.806
Anti-TPO positivity	12 (14)	12 (12.9)	0.989
Anti-TG and/or Anti-TPO positivity	18 (20.9)	19 (20.4)	0.919
Overt hypothyroidism	0 (0)	1 (1.1)	0.969
Subclinic hypothyroidism	2 (2.3)	4 (4.3)	0.750
Hyperthyroidism (Graves’ disease)	1 (1.2)	0 (0)	0.969
Euthyroidism	82 (95.3)	89 (95.7)	0.804
Subjects on Levothyroxine treatment	6 (4.7)	4 (4.3)	0.651

In the BTM group, 4.7% of patients and in the control group, 2.2% had previously undergone partial thyroidectomy due to benign thyroid disease. The two groups were statistically similar in this aspect (p=0.60) (Table-III).

The two groups were also statistically similar in respect of the frequency of patients with euthyroidism, subclinical hypothyroidism, overt hypothyroidism and hyperthyroidism (p=0.80, p=0.75, p=0.96 and p=0.96 respectively) (Table-III). Hyperthyroidism was present in only one subject among the study participants. This patient was in the BTM group and suffered from Graves’ disease. The two groups were not statistically different in respect of the frequency of patients receiving levothyroxine treatment (p=0.65). Of the patients using levothyroxine, four patients had undergone thyroidectomy and 2 were chronic autoimmune thyroiditis patients in the BTM group. In the control group, two patients were using levothyroxine because of a previous thyroidectomy operation and two patients because of chronic autoimmune thyroiditis disease.

In the BTM group, there was no bivariate correlation between HbA2 percentage and Anti-TPO and anti-TG levels.

## DISCUSSION

BTM prevalence was found to be 1.5-fold higher than the control group, which was statistically significant for the first time, in a study of 146 rheumatoid arthritis patients by Marcolongo et al. in 1975.^13^ In two studies by Montecucco, one retrospective and the other prospective, BTM prevalence was found to be increased in rheumatoid arthritis.^14^ In addition, rheumatoid arthritis incidence was shown to be increased among subjects with BTM.^15^ There can be said to be consensus on an association between arthritis and BTM.^2^,^3^ To date, rheumatological complications such as arthritis, arthropathies, joint effusions, osteoporosis, bone fractures, connective tissue disorders, pseudoxanthoma elasticum and myalgias have been defined in beta thalassemia.^3^ BTM prevalence has been found to be lower in a group of systemic lupus erythematosus subjects compared to a control group, but conversely, the disease exhibits a more serious course. Central nervous system involvement, Sjogren syndrome, serositis and renal involvement have been observed to be significantly more common among subjects with SLE and BTM.^16^ In respiratory allergic subjects with BTM and sickle cell trait, asthma prevalence has been determined to be significantly higher compared to a control group.^17^ When considered from the viewpoint of autoimmune diseases, asthma can be conceived as a type of autoimmune disease.^18^

Two mechanisms have been proposed to explain the relationship between autoimmunity and BTM. One mechanism is that the beta globin gene is located at the p15.5 locus of chromosome 11, and certain other genes that have been demonstrated to exert immunoregulatory effects, are located very close to this locus. This close gene linkage between the beta globin gene and these immunoregulatory genes might predispose subjects with BTM to autoimmunity. The other suggested mechanism is that hemorphin, a protein that suppresses inflammation and neutrophil migration, is primarily released from the beta globin chain of hemoglobin via proteolytic cleavage *in vivo*. As beta globin synthesis is reduced in BTM, it has been suggested that reduced hemorphin synthesis and/or expression might lead to autoimmunity.^2^

The above-mentioned studies prompted this examination of whether thyroid autoimmunity is increased in BTM. However, the results of this study showed that the prevalence of ATA positivity in the BTM group patients was similar to that of the control group. The two groups were also similar in terms of serum ATA levels. Asad at al. reported that TSH and FT4 were found to be higher in beta-thalassemia patients than in the control group but FT3 was similar in both groups.^12^ However, in the current study, all groups were found to be similar in respect of serum thyroid hormones. The frequency of Graves’ disease presence was also similar between the two groups. Consequently, it was determined that BTM had no significant association with autoimmune thyroid disease.

Chronic autoimmune thyroid disease may present with many autoimmune diseases including SLE, rheumatoid arthritis, scleroderma, Sjogren’s syndrome, superficial vasculitis, warm autoimmune haemolytic anemia, pernicious anaemia, immune thrombocytopenia, Type-1 diabetes mellitus, Addison’s disease, hypogonadism, hypophysitis, celiac disease, dermatitis herpetiformis, chronic active hepatitis, vitiligo, alopecia and myasthenia gravis.^19^,^20^ Therefore, subjects with known autoimmune diseases other than autoimmune thyroid disease were not included in the study. Since there is an association between stage 3 and further chronic kidney disease and thyroid autoimmunity, those patients were also excluded.^21^,^22^ Subjects with iron deficiency were not included as previous studies have stated that iron deficiency alters the HbA2 ratio in haemoglobin electrophoresis.^22^,^23^ Furthermore, celiac disease might be frequently observed in subjects with obscure iron deficiency and celiac disease itself frequently accompanies autoimmune thyroid disease.^24^ As there is a significant association between Type-2 diabetes and chronic autoimmune thyroiditis, and there were many patients with BTM and Type-2 diabetes, it was decided to include diabetes patients in the control group.^25^ Diabetes prevalence was comparable between the two groups. Smoking exerts a positive impact on thyroid autoimmunity, but the control group and the BTM group were similar in respect of the numbers of smokers and ex-smokers.^25^,^26^

Hypertension and ischemic heart disease were not considered as a reason for exclusion as these are commonly observed conditions which are not considered to have an impact on autoimmunity. Otherwise, it would have been necessary to exclude quite a large number of patients. To avoid selection bias, patients receiving anti-thyroid treatment, patients on levothyroxine treatment, and patients who had underwent thyroidectomy, were not excluded either. Autoimmune thyroid disease might not be present in each anti-TG and/or anti-TPO positive patient, especially when the titers are low.^27^ Since this is valid for both the BTM group and the control group, the study results were not considered to have been affected.

The internal disease outpatient clinics from where the study patients were recruited, function as secondary diagnosis and treatment centres. Therefore, the study patients were not subjected to a large selection and the study population might closely represent the general population. Although associations between arthritis and BTM are clear, no association was found between thyroid autoimmunity and BTM. Thyroid autoimmunity is the most common autoimmune disorder among the autoimmune diseases. The prevalence of autoimmune diseases might not be generally increased in patients with BTM, and it might be limited to arthritis. It can be suggested that ATA tests and thyroid function tests are not necessary for patients with BTM in any different way from the general population.

### Abbreviations

**BTM:** Beta thalassemia minor

**Anti-TG:** Anti-thyroglobulin antibody

**Anti-TPO:** Anti-thyroid peroxidase

**ATA:** Anti-thyroid antibody

**SLE:** Systemic lupus erythematosus

**MVC:** Mean corpuscular volume

**MCH:** Mean corpuscular haemoglobin

**FT3:** Free iodothyronine

**FT4:** Free thyroxine

**TSH:** Thyroid stimulating hormone

**Hb:** Haemoglobin

**HbA2:** Haemoglobin A2
